# Frugal Innovations in Orthopaedics

**DOI:** 10.1007/s12178-025-09985-4

**Published:** 2025-06-23

**Authors:** Shravya Kakulamarri, Charlotte F. Wahle, Lacey Smith, Sanjeev Sabharwal

**Affiliations:** 1 Institute for Global Orthopaedics and Traumatology, University of California, San Francisco, USA; 2 University of California San Francisco Benioff Children’s Hospital, 747 52nd St, Oakland, CA 94609 USA

**Keywords:** Frugal innovation, Low-cost technologies, Global health, Surgical access, Orthopaedic surgery

## Abstract

**Purpose of Review:**

Frugal innovations prioritize low-cost interventions, while keeping in mind efficacy, accessibility and scalability. Despite a scientific culture that often celebrates major financial investment and cutting-edge technologies, frugal innovations can be just as important in both low-income countries where resources are scarce as they are high income countries where the health needs of aging populations may be outpacing economic growth. We sought to comprehensively review the current state of frugal innovations in orthopaedic surgery, as well as to identify next steps as the importance of these low-cost interventions continues to grow.

**Recent Findings:**

Frugal innovation is particularly relevant in orthopaedic care as musculoskeletal interventions such as prosthetics, orthotics and surgery demand significant materials, skilled labor, and frequent follow-up. There have been numerous innovations in the recent years, including the development of low-cost intramedullary nails, bioabsorbable implants, negative-pressure wound therapy systems made from aquarium pumps, repurposed Foley catheters and nasogastric tubes for use in surgeries, among many more.

**Summary:**

Frugal innovations in orthopaedic surgery are becoming more relevant and rapidly evolving in all health-care settings as a tool to deliver value-based care to the growing needs of the population. Though many of these projects are performed on a local scale, when considered collectively, they demonstrate powerful efforts to move the needle in enhancing access to high-quality orthopaedic surgical care and reduce the burden of global musculoskeletal disability. Frugal innovations offer immense promise in reducing costs and closing the gap of access to high-quality orthopaedic care worldwide

## Introduction

Both traumatic and non-traumatic musculoskeletal conditions have been growing in incidence in low- and middle-income countries (LMICs) in recent years [1–6]. The World Bank defines high-income economies as those with more than a gross national income (GNI) per capita of $14,005, while low-income economies have a GNI per capita below $1,145 [7]. In terms of traumatic injuries, much of this growth can be attributed to increasing congestion of motor vehicle traffic coupled with high rates of motorcycle use, poor road safety and suboptimal road conditions [1, 2, 4]. On the non-traumatic side, there has been a dramatic increase in rates of obesity and metabolic disease, leading to rising rates of associated musculoskeletal pathologies such as peripheral vascular disease and osteoarthritis [3, 8]. In the context of global health as a whole, attention is often drawn to the “10–90 gap” in global health research in order to illustrate the discrepancies between disease prevalence and allocation of resources [9]. This phrase highlights the phenomenon whereby only 90% of the world’s investment in health research is aimed at addressing 10% of global health problems [9]. Thus, given the resource constraints and high level of need, the role of “frugal innovations” in healthcare, and more specifically orthopaedic care has been growing in recent years.

Frugal innovations prioritize low-cost interventions, while keeping in mind efficacy, accessibility and scalability [10–12]. Despite a scientific culture that often celebrates major financial investment and cutting-edge technologies, frugal innovations can be just as important in both low-income countries where resources are scarce as they are high income countries where the health needs of aging populations may be outpacing economic growth [12]. Frugal innovation involves being creative and resourceful to develop low-cost, widely available solutions which do not compromise their efficacy. Some common models which have been presented in the global health literature in recent years include recycling everyday materials into medical devices [13, 14] or using alternative manufacturing practices in order to reduce cost and resource burden [15–17].

Frugal innovation is particularly relevant in orthopaedic care as musculoskeletal interventions such as prosthetics, orthotics and surgery often have extremely high material and/or resource demand, skilled clinicians and frequent follow-up and imaging in order to monitor outcomes [5, 6]. In a 2008 study by Weiser et al., the lowest income 34.8% of the global population received only 3.5% of surgical procedures [18]. More specifically, in a recent large-scale study looking at trauma and orthopedic capacity across 267 hospitals in sub-Saharan Africa, the current capacity to treat orthopaedic conditions in these regions is very limited, based primarily on lack of adequate skilled personnel, training opportunities, medical facilities, and equipment [4]. Other studies have cited life- and limb-threatening delays to operative intervention in cases where patients could not afford the prohibitive costs of available orthopaedic implants [5, 19, 20]. Furthermore, there are reports of traumatic and non-traumatic amputees who are completely immobile due to insufficient access to low-cost prostheses, negatively impacting their quality-of-life [16, 21, 22].

On the pediatric side, these limitations can be even more costly as children often begin healing their traumatic musculoskeletal injuries earlier, and thus can more rapidly progress to complications such as malunion and sequalae of physeal injuries, if facing major delays to appropriate orthopaedic care including surgical intervention [23]. In a 2022 survey conducted by the Global Initiative for Children’s Surgery, the odds of incurring a catastrophic health expenditure following children’s surgery was up to 17 times greater in LIC/LMICs, drawing attention to the numerous families that often declined surgery due to inability to afford treatment [23]. Moreover, families in LICs/LMICs were significantly more likely to delay or decline surgery due to these costs. Similarly, in a geospatial study from Brazil, more limited geographical access to surgical care was associated with higher under-5 years mortality [24]. Thus, it is critical when considering surgical intervention for pediatric patients in LIC and LMICs that surgeons and other stakeholders make every effort to incorporate relevant frugal innovation practices, and reduce the prohibitive financial burden on the patients and their caretakers. While much of the existing literature focuses on the practical application of individual interventions in a local population, we seek to bridge the gap between technical implementation and the broader impact of these interventions while providing important context and considerations for effective, low-cost innovation that is scalable to the global population. Thus, we sought to comprehensively review the current state of frugal innovations in orthopaedic surgery, as well as to identify next steps as the importance of these low-cost interventions continues to grow.

### Advances in Surgical and Postoperative Care

**Fig. 1:**
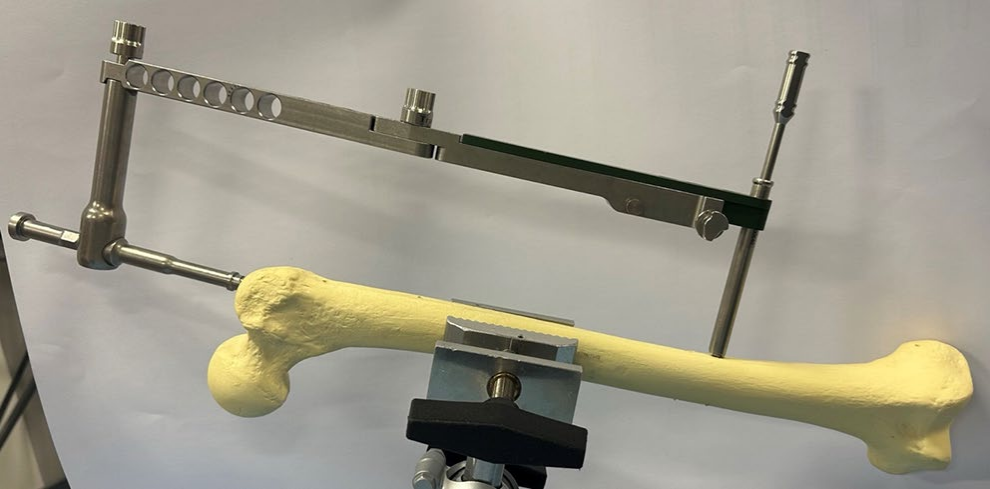
SIGN intramedullary nail system with targeting jig designed for intramedullary nail placement without intraoperative imaging. Image courtesy of SIGN Fracture Care International

The importance of low-cost implants has become increasingly apparent. In a DELPHI study evaluating the most critical resource needs for fracture and orthopaedic care in low-resource settings, various surgical implants and external fixators were widely believed to be high priority in providing optimal care [25]. While some efforts have focused on donation programs which promote the availability of high-cost implants in low-resource areas, other groups have aimed at specifically designing lower-cost implants which keep in mind local contexts and constraints. One key example of this is the Surgical Implant Generation Network (SIGN) Fracture Care International. The SIGN intramedullary nailing system was deliberately designed to be placed without the use of costly technologies such as intraoperative imaging or high-cost “fracture tables” [26] (Fig. 1). More specifically, the use of SIGN nails in low-resource settings have been successful amongst pediatric patients with femoral shaft fractures [27].

The use of bioabsorbable implants as a cost-effective option in the orthopaedic context has also been explored. Though previously viewed with skepticism due to concerns about adverse tissue reactions and mechanical weakness, ongoing research and development has attempted to address some of these concerns [28]. Certain manufacturers have developed bioabsorbable implants such as compression screws, fixation pins and plates, and mesh plates, cervical fusion systems [29, 30]. Similarly, a lab at University of Central Florida is working to create bioabsorbable implants - such as screws, pins, and rods - through magnesium composites, a naturally occurring element in the body that could minimize the risk of rejection due to higher biocompatibility [31, 32]. Though the current costs of these implants typically remain higher than traditional metal implants, with transfer of technology, scalability and the downstream decrease in costs, such as eliminating the need for revision surgeries, may ultimately make it a cost-effective choice [28].

**Fig. 2:**
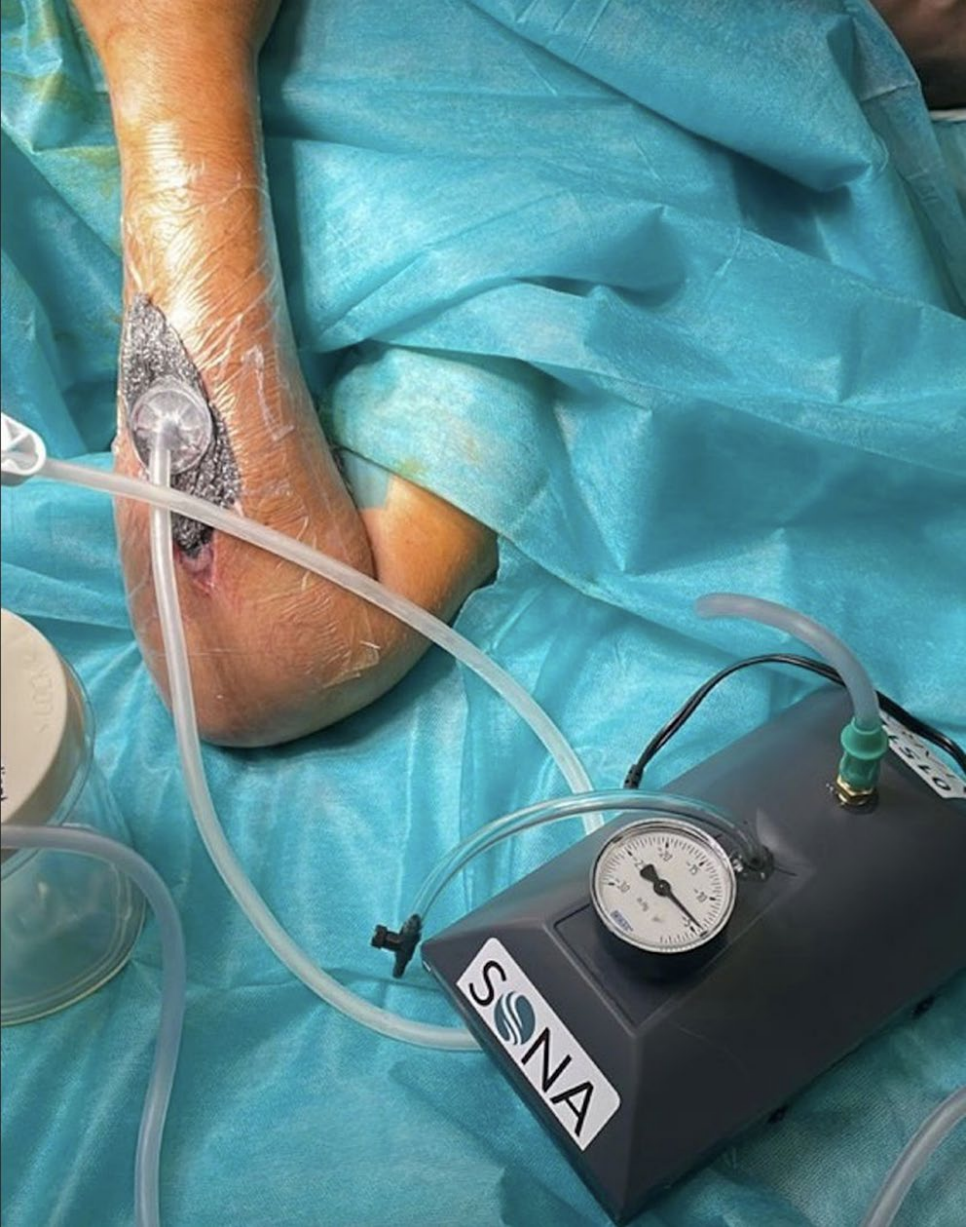
A low-cost negative pressure wound therapy (NPWT) device in use, developed for resource-limited settings. Image courtesy of Dr. Kiran Agarwal-Harding

Additionally, negative pressure wound therapy (NPWT) is often used in the management of open fractures and other soft tissue defects that are at high risk of infection or delayed healing [33]. These devices also aid in wound closure, while reducing edema. Historically, the cost of NPWT devices has made them prohibitive in low-resource settings. However, researchers in Haiti, Brazil, and the Philippines have explored low-cost NPWT devices made from locally available materials like aquarium aerator pumps [34–36]. Amlani et al. developed a similar low-cost NPWT utilizing aquarium aerator pumps, pressure gauges, and bleed valves, costing them 100 USD, which is substantially less expensive than their commercially available counterparts manufactured in high-income countries [33] (Fig. 2). They evaluated the efficacy of their locally manufactured NPWT device in Cameroon and demonstrated improvement in wound margins in 89% of patients with open wounds and fractures across their body, ranging from the upper extremity, to the torso, to the lower extremity [4]. Additionally, 80% of patients achieved wound closure [33]. Aiming to address a similar need, Su et al. developed and investigated the use of a waterproof, low-cost dressing system for patients undergoing total hip arthroplasty [37]. The current standard for dressings is aseptic gauze and plastic tape. Postoperative wound complications are a large predictor of periprosthetic infection; this risk can be minimized with proper dressings. Their team developed calcium alginate dressings paired with IV3000 film, which can form a gel-like consistency and ensure moisture around the wound [37]. Preliminary results from efficacy trials indicated that there are fewer dressing changes required for patients [37]. However, further testing must be conducted to understand the direct impact on infection rates and its applicability in low-resource environments.

### Innovations in Amputation Prostheses

Amputations often occur in LMICs due to traffic accidents, complex wounds, and deep and persistent infections [15]. According to the World Health Organization, 30 million people need prostheses and orthotics [38]. The 2030 Agenda for Sustainable Development has outlined the importance of assistive technologies as essential for individuals with disabilities, building on the principle of “leaving no one behind.” [39] Prostheses play a critical role in addressing disability by helping patients regain mobility and independence. Given the high barrier of costs and resources required, the availability prosthetic services is generally inadequate in LMICs [40, 41]. Providing prosthetic support in LMICs involves a number of factors unique to the context, including rural environments, extreme and rugged local climate and terrain, limited access to transportation and health care facilities. The implication of amputation in many LMICs has additional economic and cultural considerations, such as context-specific attitudes towards disability and the reintegration of amputees into the society and local community. Further, ensuring longitudinal prosthetic service delivery including regular maintenance and fittings for optimal function is often difficult in rural and resource-limited settings [2]. In fact, when patients are not able to access the resources necessary to keep prostheses in optimal condition, they have worse functional outcomes and quality of life compared to individuals that have the proper resources to maintain their prostheses, essentially allowing for optimally functioning prostheses [42, 43]. Ultimately, in-country development and manufacturing of these prosthetic devices enables that they are accessible, affordable, and better tailored to the needs of local populations undergoing amputations.

A growing number of organizations and companies across sub-Saharan Africa have been working to develop low-cost, accessible prostheses. Much of the ongoing work in this field is exploring the feasibility of 3D printed limb prostheses, with the primary goal of decreasing the high cost of commercially manufactured conventional prostheses. Additionally, 3D printed customized prosthetic limbs have the potential to fit more accurately and comfortably than the conventional prosthetic limbs developed manually with plaster of Paris and molds of the residual limb. Current 3D scanning technologies can more accurately digitize the patients’ limbs. Furthermore, manual production of prostheses is a time-intensive process, requiring weeks to months, from initial consultation, to plaster cast and negative plaster mold, to melting and positive plaster mold, followed by trimming and adjustment [44].

Several countries across sub-Saharan Africa have reported successful pilot projects involving 3D printed prosthetic devices. In a 2021 study from Sierra Leone, a team created 3D printed, low-cost transtibial prostheses and sockets. Results from the study showed that patients were still wearing the prosthesis at an early (6 weeks) follow-up and all patients had reached their personal goals in terms of mobility [15]. Similarly, in Uganda, a trained orthopedic technician has built affordable prosthetics using locally available recycled plastics and is planning to open another workshop in western Uganda, on the border of the Congo to cater to victims of war [45]. Another hospital in Uganda, Comprehensive Rehabilitation Services hospital (CoRSU) also provides 3D printed limbs for children through a Canadian nonprofit organization [46–48]. In neighboring Rwanda, the United Nations Development Programme (UNDP) partnered with a local rehabilitation center to launch a 3D computer-aided design (CAD) for manufacturing low-cost orthotics and prosthetics [49]. Additionally, EmpowerAbility through Bridging Afrika, an organization that aims to leverage technology and innovation to target social challenges, is planning to produce affordable, locally produced 3D printed prostheses [50]. Similarly, in Malawi, the Lilongwe Institute of Orthopaedics and Neurosurgery (LION) has partnered with 500 Miles, an organization from the UK that develops prosthetics and orthotics and the Malawi Ministry of Health. Patients are asked to contribute whatever they are able to afford, and the rest of the cost is subsidized by 500 Miles. Though many of these projects represent local pilots, E-NABLE is an online community of volunteers with access to 3-D printers that aims to expand access to free and low-cost prosthetic upper limb devices for individuals in LMICs [51]. The designs are open-source and were designed by the volunteers themselves. To date, 40,000 volunteers in over 100 countries have delivered free hands and arms to 10,000–15,000 recipients.

### Repurposed Materials and Techniques

**Fig. 3:**
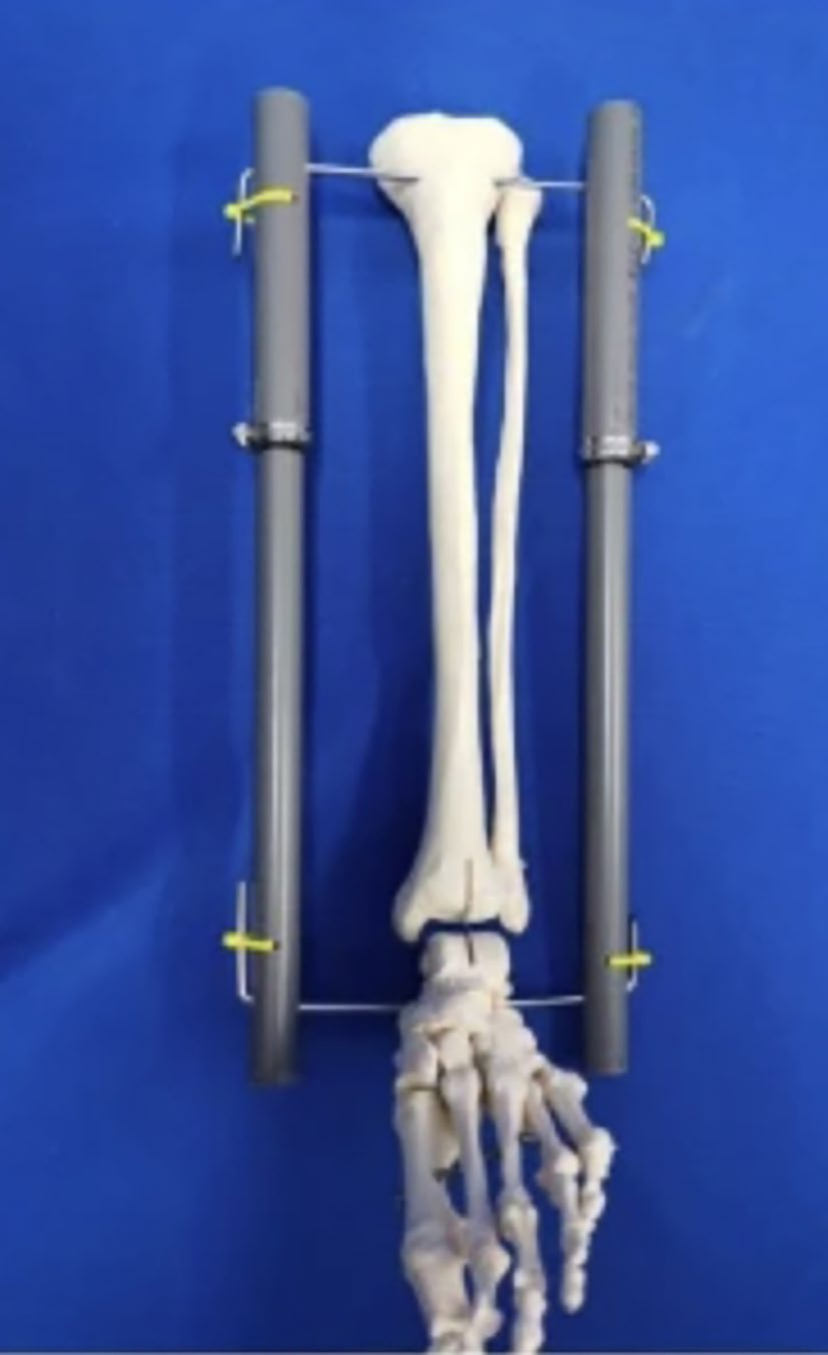
PVC pipe-based bilateral traction device used in lower limb fractures. Image courtesy of Dr. Shuye Yang and co-authors

**Fig. 4:**
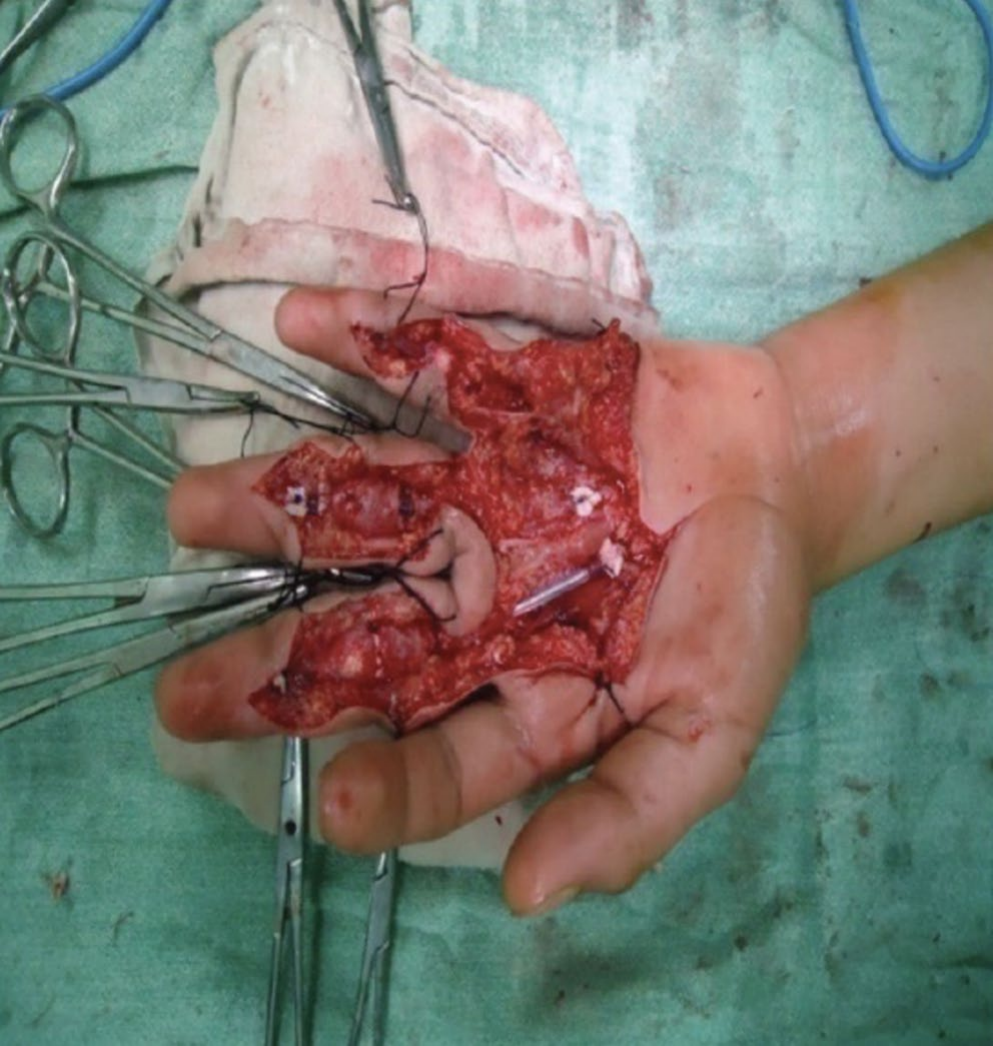
Silicone Foley catheter used as a low-cost alternative to Hunter’s rod in staged flexor tendon reconstruction of the hand. Image courtesy of Dr. Tawheed Ahmad and co-authors

Another area of frugal innovation which carries great promise is the recycling or repurposing of common or low-cost materials into needed orthopaedic tools or devices [52]. One example of this is the use of polyvinyl chloride (PVC) pipes as an adjustable bilateral traction device for lower limb fractures [13] (Fig. 3). In this study, 36 patients with lower limb fractures were treated with a traction device made from locally available PVCs, offering a low cost, lightweight alternative to traditional traction devices [13]. This frugal innovation has promise in pediatric orthopaedic surgery where traction devices are often used for the gradual correction of congenital deformities or limb length differences in addition to orthopaedic trauma, especially in LMICs. Another low-cost substitute for similar applications is bamboo and other plant-based materials [53]. Recent work by Sujithra et al. used a novel green pea pod lignin as well as hybridized pod sheath fibre-bamboo epoxy composite for amputation prostheses [53]. In the field of reconstructive hand surgery, specifically flexor tendon repair, materials like the Hunter silicone rod are considered the “gold standard”. However, the Hunter rod is constructed from barium-impregnated silicone elastomer with a woven polyester core that reportedly costs approximately $250 [54]. In resource-limited settings, surgeons have explored feasible alternatives to the Hunter rod that can serve as the temporary implant in the first stage of the tendon repair procedure. A team of surgeons in India investigated the use of a silicone Foley catheter as an alternative to Hunter’s rod in surgeries for seventy digits [55] (Fig. 4). Using the Strickland-Glogovac grading system for evaluating range of motion 70% were reported as “excellent” and 20% were “good.” [55] Similarly, a group in Turkey studied the use of nasogastric tubes as a low-cost substitute for Hunter’s rods in surgeries for twenty-four severed digits [56]. Excellent results were observed in 58.3% of patients and good results in 25% [56]. Another group in India has also reported satisfactory outcomes with the use of ordinary PVC feeding tubes as an alternative in surgeries on eight severed digits [57]. In summary, materials like Foley catheters, nasogastric tubes, and PVC feeding tubes have shown comparable functional outcomes to Hunter’s rod, exemplifying the potential for repurposed innovation geared towards low-resource settings.

Beyond repurposing raw materials, there has also been documented success in repurposing surgical tools or technologies from their original purpose to fulfill a new need. One example of this is the Ilizarov technique and fixator [58, 59]. This technology was originally used to treat tibial nonunion by employing opposing external compressive forces. However, it was later discovered that callus could also form when moving these forces in the opposite direction (pulling apart rather than pushing together) [60]. In the years since, this technology has been employed for a variety of limb-deformity and limb-lengthening purposes, including pediatric patients, thus offering a creative biologic solution to a challenging problem [60, 61]. Additionally, there are various tools that are used intraoperatively for the repair of small joints such as for metatarsophalangeal joint instability. However, these specialized suture passing devices and needles are expensive, ranging from $25 up to $700 [62, 63]. Kindred et al. successfully used an angiocatheter needle to guide repair of the plantar plate by guiding suture placement [64]. Angiocatheter needles are much more affordable and are readily available in a hospital setting, allowing for easy repurposability of these tools to aid in surgery. Similarly, the Arbutus DrillCover System promotes the repurposing of commercial power drills to address the high costs of orthopaedic drill systems [65]. The DrillCover System provides a waterproof, autoclave-safe cover that can be sterilized up to 75 times which repurposes a relatively less-expensive and more readily available commercial power drill as a surgical tool. It also comes with a surgical chuck that connects to standard orthopaedic bits. Selhorst et al. evaluated the use of the DrillCover System in the United States and noted that it was noninferior to conventional surgical drills regarding infections at the site of skeletal traction pins [66]. Innovation through repurposing is inherently low cost, as it ideally involves no new spending, but rather taking an existing material or tool and using it in a novel way to solve a different problem.

### Expanding Access: High-Quality, High-Volume, Low-Cost Orthopaedic Care Models

Finally, apart from low-cost physical and technical innovations, improving healthcare on a systems level can help create sustainable and locally relevant health-delivery models. The high-quality, high-volume, and low-cost model has proven to be a powerful strategy for delivering specialized healthcare in low-resource settings. This model functions through the cross-subsidization methodology, balancing paying and non-paying patients to ensure a financially solvent system. There are programs that are utilizing this methodology in the field of orthopaedics, such as the Jaipur Foot Organization, Narayana Health, and CURE [67–69].

## Conclusion

Frugal innovations in orthopaedic surgery are becoming more relevant and rapidly evolving in all health-care settings as a tool to deliver value-based care to the growing needs of the population. Though many of these projects are performed on a local scale, when considered collectively, they demonstrate incredible efforts to move the needle in enhancing access to high-quality orthopaedic surgical care and reduce the burden of global musculoskeletal disability. Through the development of new technologies such as low-cost prosthetic devices, surgical implants and dressings, as well as the repurposing of existing materials and tools, there is potential to bridge the gap to access a variety of life-changing orthopaedic treatments, including surgical procedures, orthoses and prostheses. Among these, there are three innovations that stand out most to us and display significant potential in terms of impact and scalability: the SIGN intramedullary nailing system, low-cost NPWT devices, and the Arbutus DrillCover system. These solutions reflect the ingenuity and practical value of frugal innovation in real-world settings.

Though many of the innovations mentioned show great promise, many of these technologies are relatively novel and have only been recently implemented. Thus, it is important to continue monitoring for roadblocks and complications over time to ensure equality and safety in their use and expanding applications. Additionally, while much of the focus of this review has been geared towards low-resource environments where costs may be prohibitive, they also offer tremendous opportunity to optimize costs and reduce unnecessary waste in higher-resource environments. As frugal innovations aim to reduce costs while maintaining quality of care in low-resource settings, there is a lot of potential for these technologies and methodologies to be adopted in higher-resource settings with the same purpose and goals, ultimately decreasing healthcare costs and addressing disparities worldwide. In conclusion, frugal innovations offer immense promise in reducing costs and closing the gap of access to high-quality orthopaedic care worldwide.

## Key References

- Dworkin M, Woolley PM, Shahab F, et al. Access to Orthopaedic Devices in Low and Middle-Income Countries: Challenges and Opportunities. J Bone Joint Surg Am. Published online March 27, 2025. doi:10.2106/JBJS.24.00997
-
  - Dworkin et al. (2025) eloquently discusses the challenges and opportunities surrounding sustainable access to orthopaedic devices in low- and middle-income countries, emphasizing the critical measures needed within these countries to ensure proper regulation and adoption of devices.
- Chokotho L, Jacobsen KH, Burgess D, et al. Trauma and Orthopaedic Capacity of 267 Hospitals in East Central and Southern Africa. Lancet. 2015;385 Suppl 2:S17. doi:10.1016/S0140-6736(15)60812-1
-
  - Chokotho et al. (2015) presents a detailed assessment of 267 hospitals in East Central and Southern Africa, highlighting the limited capacity to treat trauma and orthopaedic conditions– particularly due to shortages in manpower, equipment, and facilities.
- Sekimpi P, Okike K, Zirkle L, Jawa A. Femoral fracture fixation in developing countries: an evaluation of the Surgical Implant Generation Network (SIGN) intramedullary nail. J Bone Joint Surg Am. 2011;93(19):1811–1818. doi:10.2106/JBJS.J.01322
-
  - Sekimpi et al. (2011) evaluates the use of the SIGN intramedullary nail– a novel innovation– for femoral fracture fixation in developing countries, providing evidence on the effectiveness and practicality of affordable, scalable orthopaedic implants in low-resource settings.
- Prime M, Attaelmanan I, Imbuldeniya A, Harris M, Darzi A, Bhatti Y. From Malawi to Middlesex: the case of the Arbutus Drill Cover System as an example of the cost-saving potential of frugal innovations for the UK NHS. BMJ Innov. 2018;4(2):103–110. doi:10.1136/bmjinnov-2017-000233
-
  - Prime et al. (2018) showcases the Arbutus Drill Cover System, demonstrating how commonly available tools in high and low-resource clinical settings can be effectively repurposed without compromising surgical outcomes.
- Selhorst S, O’Toole RV, Slobogean GP, et al. Is a Low-Cost Drill Cover System Noninferior to Conventional Surgical Drills for Skeletal Traction Pin Placement? J Orthop Trauma. 2021;35(11):e433-e436. doi:10.1097/BOT.0000000000002064
-
  - Selhorst et al. (2021) builds upon the work of Prime et al. (2015) by providing evidence that the low-cost drill cover system is noninferior to conventional surgical drills for skeletal traction pin placement, highlighting the feasibility of adopting affordable technologies in higher-resource environments.

## Data Availability

No datasets were generated or analysed during the current study.
